# Supplementary material for: Docking and Electronic Structure of Rutin, Myricetin, and Baicalein Targeting 3CLpro
Source: Int J Mol Sci. 2023 Oct 12;24(20):15113. doi: 10.3390/ijms242015113 (PMC10606270; doi:10.3390/ijms242015113)
Supplement: Supplementary file 1 [file ijms-24-15113-s001.zip › ijms-2603896-supplementary.pdf]

Table S1: Torsion angles (degrees) of rutin, myricetin, and baicalein optimized using CAM-B3LYP/def2TZV level using IEFPCM. The calculated dihedral angles are τ (**O1-C2-C1′-C6′**), ω (**C9-O1-C2-C3**), ϕ (**C10-C4-C3-C2**), and, for rutin, δ (**C5″-C6″-O6″-C1‴**). Vacuum values are in parenthesis.

	$\boldsymbol{\tau}$	$\boldsymbol{\omega}$	$\boldsymbol{\phi}$	$\boldsymbol{\delta}$
Rutin	147.41<br>(168.95)	−2.73<br>(0.63)	6.69<br>(7.20)	155.12<br>(177.82)
B3LYP/6−311G [22]	−162.03	-	-	-
B3LYP/6-311G[8]	142.00	-	-	173.0
Quercetin [13]	169.59	7.27	7.13	169.59
Myricetin	−168.90<br>(−170.58)	0.32<br>(0.54)	−2.47<br>(−2.19)	
[51]	±60 and ±120	-	-	-
[52]	180.0	-	-	-
B3LYP/6-31++G(d,p) and M062X/6-31++G(d,p) [24]	180.0	-	0.00	-
PDB 7DPP [9]	131.82	0.54	−2.19	-
Baicalein	−157.97<br>(−159.99)	−0.08<br>(−0.45)	−1.79<br>(−1.76)	-
PDB 4X2A [53]	142.32	−0.03	−0.02	-
PDB 4X2A [53]	136.26	0.01	0.04	-
